# Extending precision medicine tools to populations at high risk of type 2 diabetes

**DOI:** 10.1371/journal.pmed.1003989

**Published:** 2022-05-19

**Authors:** Shivani Misra, Jose C. Florez

**Affiliations:** 1 Division of Metabolism, Digestion & Reproduction, Faculty of Medicine, Imperial College London, London, United Kingdom; 2 Department of Diabetes, Endocrinology & Metabolism, Imperial College Healthcare NHS Trust, London, United Kingdom; 3 Diabetes Unit and Center for Genomic Medicine, Massachusetts General Hospital, Boston, Massachusetts, United States of America; 4 Programs in Metabolism and Medical & Population Genetics, Broad Institute of Harvard and Massachusetts Institute of Technology, Cambridge, Massachusetts, United States of America; 5 Department of Medicine, Harvard Medical School, Boston, Massachusetts, United States of America

## Abstract

In this Perspective, Shivani Misra and Jose C Florez discuss the application of precision medicine tools in under-represented populations.

People of South Asian ancestry carry a 3-fold higher risk of developing type 2 diabetes (T2D) than white European individuals [1], with the disease typically manifesting a decade earlier [2] and at a leaner body mass index (BMI) [3]. The South Asian population is often considered as a uniform group, but significant heterogeneity in the prevalence of T2D and its phenotype manifestations across south Asia exists, with a higher prevalence in those from Bangladeshi and Pakistani communities [4]. Genome-wide association studies (GWAS) have not fully explained the excess risk observed in South Asian individuals [5,6], and attention has turned to strategies through which genetic information may be leveraged for clinical benefit, such as generating an aggregate of weighted single nucleotide polymorphisms (SNPs) that capture the overall genetic burden for a trait into a polygenic score (PS) (sometimes described as a polygenic risk score) [7]. However, constructing a PS remains challenging in populations that are underrepresented in GWAS.

In the accompanying article in *PLOS Medicine* [8], Hodgson and colleagues investigate the use of a PS to predict T2D in the Genes & Health (G&H) cohort, addressing a key knowledge gap in the applicability of such tools in underrepresented ethnicities. G&H is a pioneering community-based cohort of approximately 48,000 participants of predominantly British Bangladeshi and Pakistani heritage combining genetic and longitudinal electronic healthcare record data. They first assessed the transferability of known T2D genetic risk loci in G&H and constructed a PS using variants from a multi-ancestry GWAS, adjusting the scores for Pakistani and Bangladeshi individuals and selecting the one with the highest odds for prediction. This score was then integrated with 3 versions of a clinical model (QDiabetes) to predict T2D onset over 10 years in 13,642 individuals diabetes free at baseline. The authors show that incorporation of a PS with QDiabetes provided better discrimination of progression to T2D, especially in those developing T2D under 40 years of age and in women with a history of gestational diabetes. Finally, they incorporated the PS into cluster analyses of baseline routine clinical characteristics, replicating clusters defined in European populations and identifying a cluster resembling a subgroup of severe insulin deficiency. This study significantly advances the field on the transferability of PSs, reproducibility of T2D clusters, and clinical translation of these findings to precision medicine for diabetes.

## Transferability of PSs and T2D clusters to other ethnicities

The generation of ethnicity-specific T2D PS for every subethnic group remains aspirational, as the large sample sizes needed to do this robustly are prohibitive (the genetic ancestry of the Indian subcontinent, for example, is more diverse than the whole of Europe [9]). Thus, strategies to utilise existing scores derived from other populations, or leveraging multi-ancestry GWAS, have predominated. Attempts to apply a PS derived in a population of one ancestry to another ethnic group have shown variable performance [10,11]. Indeed, a lower percentage of GWAS loci than expected were replicated in the present study, and several loci previously identified in another South Asian-specific GWAS also failed to replicate [5]; although this may be related to power, it also demonstrates the heterogeneity within South Asian populations, arguably more relevant in cultures where consanguineous unions are prevalent.

Previously described data-driven diabetes clusters [12] have been partially reproducible in other ethnicities including Indian cohorts [13]. Despite the absence of specific biomarker data (C-peptide and pancreatic autoantibodies), the present study was able to identify a probable insulin-deficient cluster on the basis of a high PS reflecting variants associated with beta-cell dysfunction and leaner BMI. It remains unclear whether data-driven clustering approaches stratifying the T2D population offer benefit over and above routine care.

## What is the clinical benefit in a higher-risk group?

Precision medicine strategies such as the use of a PS in T2D have predominantly focused on European populations, even though South Asian populations account for a significant proportion of all T2D cases. A strength of Hodgson and colleagues’ study is the identification of a specific subset of individuals where the PS may add value; early-onset T2D progresses rapidly to insulin treatment and carries a higher risk of microvascular and macrovascular complications and earlier mortality. The present study poses an interesting research question: Could identifying people earlier in life through an elevated PS attenuate progression to T2D through lifestyle interventions or more aggressive cardiovascular risk reduction?

## Future directions

The study by Hodgson and colleagues has some limitations: Because South Asians are underrepresented in GWAS, power to discover and select relevant SNPs was finite. Given the unique cohort assembled, there was also no opportunity to replicate prediction and clustering in another cohort. The linked medical records yielded incomplete clinical data to robustly define and replicate previous phenotype clusters and conclusively demonstrate diabetes subtypes. Finally, the risk of undiagnosed diabetes in some control participants cannot be excluded.

Despite these acknowledged limitations, the findings show a clear pathway for future investigations. First, larger GWAS are needed in Pakistani, Bangladeshi, and other South Asian and non-European populations, to enhance the robustness of PS and their portability [4]. Additionally, more investigations are needed to understand the effect of PS in clinical practice. This should occur individually (e.g., qualitative analysis of the effects of genetic information on patient behaviour and lifestyle) and at a clinical pathway level (i.e., whether the PS changes management over and above routine clinical metrics, particularly in resource-limited healthcare settings). Finally, the application of process-specific PS (scores that cluster SNPs based on physiological associations) has huge potential to interrogate the biology underlying T2D [14].

Hodgson and colleagues provide compelling evidence that precision medicine tools can be applied meaningfully in hitherto underrepresented populations, and building resources such as the G&H cohort is a valuable endeavour. They provide knowledge upon which clinical studies that test utility of PS in South Asians can be designed. Given the higher prevalence of T2D in South Asians, such studies are critical. However, their study also illustrates the challenges of studying underrepresented ethnicities. Overcoming such challenges may yield not only genetic discovery, but also therapeutic opportunities for vulnerable populations.

## Abbreviations:

BMI: body mass index
G&H: Genes & Health
GWAS: genome-wide association studies
PS: polygenic score
SNP: single nucleotide polymorphism
T2D: type 2 diabetes
